# Antibiotics at birth and later antibiotic courses: effects on gut microbiota

**DOI:** 10.1038/s41390-021-01494-7

**Published:** 2021-04-06

**Authors:** Sofia Ainonen, Mysore V Tejesvi, Md. Rayhan Mahmud, Niko Paalanne, Tytti Pokka, Weizhong Li, Karen E Nelson, Jarmo Salo, Marjo Renko, Petri Vänni, Anna Maria Pirttilä, Terhi Tapiainen

**Affiliations:** 1 PEDEGO Research Unit and Medical Research Centre Oulu, University of Oulu, Oulu, Finland; 2 Ecology and Genetics, Faculty of Science, University of Oulu, Oulu, Finland; 3 Department of Pediatrics and Adolescent Medicine, Oulu University Hospital, Oulu, Finland; 4 J. Craig Venter Institute, La Jolla, CA USA; 5 J. Craig Venter Institute, Rockville, MD USA; 6 Department of Paediatrics, University of Eastern Finland and Kuopio University Hospital, Kuopio, Finland; 7 Biocenter Oulu, University of Oulu, Oulu, Finland

## Abstract

**Background:**

Intrapartum antibiotic prophylaxis (IAP) is widely used, but the evidence of the long-term effects on the gut microbiota and subsequent health of children is limited. Here, we compared the impacts of perinatal antibiotic exposure and later courses of antibiotic courses on gut microbiota.

**Methods:**

This was a prospective, controlled cohort study among 100 vaginally delivered infants with different perinatal antibiotic exposures: control (27), IAP (27), postnatal antibiotics (24), and IAP and postnatal antibiotics (22). At 1 year of age, we performed next-generation sequencing of the bacterial 16S ribosomal RNA gene of fecal samples.

**Results:**

Exposure to the perinatal antibiotics had a clear impact on the gut microbiota. The abundance of the Bacteroidetes phylum was significantly higher in the control group, whereas the relative abundance of *Escherichia coli* was significantly lower in the control group. The impact of the perinatal antibiotics on the gut microbiota composition was greater than exposure to later courses of antibiotics (28% of participants).

**Conclusions:**

Perinatal antibiotic exposure had a marked impact on the gut microbiota at the age of 1 year. The timing of the antibiotic exposure appears to be the critical factor for the changes observed in the gut microbiota.

**Impact:**

- Infants are commonly exposed to IAP and postnatal antibiotics, and later to courses of antibiotics during the first year of life.
- Perinatal antibiotics have been associated with an altered gut microbiota during the first months of life, whereas the evidence regarding the long-term impact is more limited.
- Perinatal antibiotic exposure had a marked impact on the infant’s gut microbiota at 1 year of age.
- Impact of the perinatal antibiotics on the gut microbiota composition was greater than that of the later courses of antibiotics at the age of 1 year.

## Introduction

Neonates are routinely exposed to antibiotics at birth and in the perinatal period.^1,2^ Intrapartum antibiotic prophylaxis (IAP) is commonly used to prevent severe neonatal bacterial infections, sepsis, and meningitis caused by the Group B *Streptococcus* (GBS) and *Streptococcus agalactiae*; a substantial use of IAP has reduced the incidence of the early-onset GBS sepsis by 50–80%.^3,4^ Universal antenatal screening of pregnant women for GBS colonization, a widely recommended policy in many developed countries, has shown that 20–30% of pregnant women and their newborn infants are exposed to IAP.^5–7^ In addition, an estimated 2–5% of newborns are exposed to the empirical postnatal intravenous antibiotics used to treat suspected neonatal sepsis.^8^

Several studies have indicated that both IAP and empirical antimicrobial therapy after birth influence the subsequent composition and biodiversity of the gut microbiota in vaginally delivered infants during the first few months.^9–11^ However, it is not known whether these changes persist beyond early infancy. Furthermore, infants and young children frequently receive courses of oral antibiotics for common childhood infections.^12,13^ In previous epidemiological studies, early exposure to oral antibiotics has been associated with subsequent asthma, allergic diseases, overweight, inflammatory bowel disease, and celiac disease in childhood.^14–19^ In a previous study, oral macrolide use was associated with long-term alterations in the gut microbiota in daycare children.^20^

In our earlier prospective, controlled study of vaginally delivered newborn infants, perinatal antibiotic exposure rapidly changed the gut microbiota during the first week of life, with changes persisting up to the age of 6 months.^11^ Our hypothesis was that the observed differences vanish as the infants grow older and are exposed to courses of antimicrobials. We have now followed up the cohort and compared the impacts of perinatal antibiotics and courses of antibiotics on the composition of the gut microbiota at 1 year of age.

## Methods

### Study design and study population

**Fig. 1:**
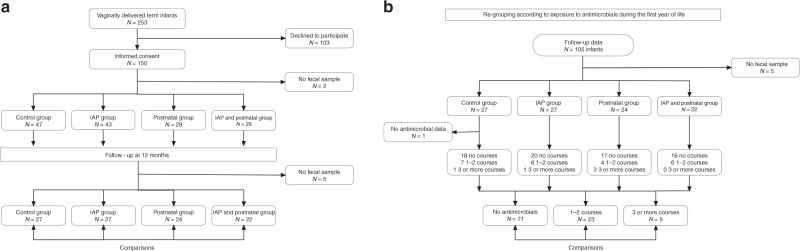
Study design. IAP refers to intrapartum antibiotic prophylaxis. **a** Flowchart and comparisons at 1 year of age according to the perinatal antibiotic exposure. **b** Flowchart and comparisons according to exposure to courses of antibiotics after the perinatal period.

The participants in this prospective, controlled cohort study (Fig. 1) were vaginally delivered term infants born between February 2014 and June 2015 at Oulu University Hospital, Oulu, Finland. The parents of all the recruited infants gave their written informed consent. Only families with decent Finnish language skills were included. Infants born with severe congenital disorders were not included in the study. None of the children had a neurological or developmental problem at the age of 1 year. We have earlier reported the influence of perinatal antibiotics on the early gut microbiota in the same cohort up to 6 months of age.^11^ We now collected data on antimicrobial consumption after the perinatal period and obtained a fecal sample at the age of 1 year. The research plan was reviewed and approved by the Regional Ethics Committee of the Northern Ostrobothnia Hospital District, Oulu University Hospital, Oulu, Finland (decision number EETTMK 76/2013).

### Antibiotic exposures and samples

The participating vaginally delivered infants were divided into four groups based on their perinatal antibiotic exposure. Detailed perinatal antibiotic exposures were collected from comprehensive hospital medical records by the study physician. The *control group* was not exposed to IAP during delivery, or to postnatal antibiotics during the first week of life, or to any maternal antibiotics within 1 week before delivery, the *IAP group* received only IAP, the *postnatal group* was exposed to empirical postnatal intravenous antibiotics within 24 h after birth, and the *IAP* + *postnatal group* received both IAP and postnatal intravenous antibiotics. Inclusion criteria to *IAP group* and *IAP* + *postnatal group* was that maternal antibiotic prophylaxis was administered 2–24 h before the birth and in the *postnatal* group and *IAP* + *postnatal* group infants had to receive postnatal antibiotics in 24 h after the birth to be included in the study. All the infants exposed to postnatal antibiotics received a *Lactobacillus reuteri* probiotic product with a daily dose of 10^8^ colony-forming units during the first week of life until discharge. The families then collected a fecal sample from the infant’s diaper or potty at 12 months of age and sent these to the laboratory for storage at −20 °C and later analysis.

For oral antibiotic courses, the study nurse contacted all families at the age of 6 months and again at the age of 12 months either by a web-based survey sent by email or by telephone contact. The timing of oral antibiotic courses, including drug names and indications, were recorded (Table 1). The infants were then regrouped based on their antimicrobial consumption after the perinatal period: (1) no courses of antibiotics, (2) one or two courses of antibiotics, and (3) three or more courses of antibiotics.

**Table 1:** Antibiotic exposures.

	Control, *N* = 27	IAP, *N* = 27	Postnatal, *N* = 24	IAP + postnatal, *N* = 22
Antibiotics during pregnancy, *N* (%)	7(26)	7(26)	4(17)	9(41)
Intrapartum antibiotics (IAP), *N* (%)				
Penicillin G		25 (93)		13 (59)
Cefuroxime		2 (7)		3 (14)
Clindamycine		0 (0)		4 (18)
Penicillin + cefuroxime		0 (0)		2 (9)
No. of intrapartum antibiotic doses, *N* (%)				
1		10 (37)		10 (45)
2		12 (44)		7 (32)
3		2 (7)		3 (14)
4		3 (11)		2 (9)
Postnatal antibiotics started, *N* (%)				
Penicillin G			3 (13)	4 (18)
Penicillin G + tobramycin			21 (88)	18 (82)
No. of antibiotic courses in the first 12 months, *N* (%)				
β-Lactams^a^	9 (20)	10 (23)	10 (23)	6 (14)
Macrolides^b^	2 (5)		3 (7)	
Sulfatrimethoprime			2 (5)	1 (2)
Unknown			1 (2)	
Time between the last antibiotic course and 12-month sample (months), median (range)	2 (8)	2 (4)	3 (8)	3 (7)
Time between the last antibiotic course and 12-month sample (months), mean (SD)	3.1 (2.8)	2.9 (1.5)	3.6 (2.9)	3.8 (2.7)

*IAP* intrapartum antibiotic prophylaxis.

^a^β-Lactams include amoxicillin with and without clavulanic acid, ampicillin, cephalexin, and ceftriaxone.

^b^Macrolides include azithromycin and clindamycin.

### DNA extraction, PCR, and microbiota analysis

DNA extraction from fecal samples was performed using the QIAamp Fast DNA stool mini kit according to the protocol provided by the manufacturer. The final product was eluted to 50 µl to increase the DNA yield further. The DNA concentration was measured using Nanodrop. The samples were stored at −20 °C until used. We have previously reported the PCR reagents and cycling conditions and analyses of the PCR from the fecal sample in detail.^11^ Universal R926trP1 and F519 primers with unique barcodes were used to amplify a portion of the bacterial 16S ribosomal RNA (rRNA) gene. PCR was performed on a Veriti 96-Well Thermal Cycler from Applied Biosystems. The reactions were performed in triplicate for all the samples. The PCR products were purified with an AMPure XP PCR clean-up kit from Agencourt Bioscience. DNA concentrations were measured with a Bioanalyzer DNA chip from Agilent Technologies. The samples were pooled in equimolar amounts and the pooled samples were purified again with the AMPure XP kit. The DNA concentration in the final product was measured with a Quant-iT PicoGreen dsDNA Assay kit from Thermo Fisher Scientific. The Ion PGM Hi-Q View OT2 kit for a 400 bp protocol, the Ion PGM Hi-Q View Sequencing kit, and Ion 316 v2 chips were used for sequencing, which was performed with an Ion Torrent PGM instrument.

### Bioinformatics analysis

The next-generation sequencing data for the bacterial 16S rRNA gene were processed by the J. Craig Venter Institute (JCVI) 16S annotation pipeline, which was integrated using programs from the Uparse,^21^ Mothur,^22^ and Biom^23^ packages. Raw reads were then filtered using the usearch fastq_filter command (maximum expected error threshold at 1.0). Unique reads were identified and singletons removed were by means of usearch derep_fulllength and sortbysize with default parameters. The operational taxonomic units (OTUs) were then clustered by 97% identity with usearch cluster_otus command, which also performed chimera filtering. The OTUs were annotated by the Mothurclassify.seqs command with the default parameters, using SILVA^24^ as the reference database. The OTU feature table was converted to biom format using the biom tool. The data were then rarefied to 3300 reads per sample and the alpha and beta diversity (using principal coordinate analysis) was assessed in QIIME2.^25^ The illustrated microbiota was drawn using the Krona visualization tool.^26^ All the raw sequences were submitted to GenBank sequence reads archives (SRA) with the Bioproject SRA accession number: PRJNA605735.

### Statistical analysis

We first analyzed the associations between perinatal antibiotics and the gut microbiota at the age of 12 months, followed by those between the courses of antimicrobials during the first year of life and the gut microbiota at 12 months. The relative abundances of the main bacterial phyla and genera, indices of alpha diversity, and number of OTUs were compared. The independent samples *t* test was used first to compare the relative abundances and biodiversity indices between the control group and the group of all those exposed to perinatal antibiotics (IAP, postnatal, IAP + postnatal combined) to ensure sufficient statistical power. In a similar manner, we then used the *t* test for comparisons between those not exposed to antimicrobials and the group exposed to at least one course of antimicrobials (≥1 course). Comparisons between all three perinatal antibiotic groups and the control group were performed first using an analysis of variance followed by post hoc tests with Tukey’s HSD (honestly significant difference) or Games–Howell correction for multiple comparisons. The same was done for the groups formed in terms of antimicrobial courses in the first year of life (none, 1–2 courses, and ≥3 courses). We then performed the linear mixed model adjusted for the different antibiotic exposure groups to control the possible confounding effect of the antibiotic exposure after the perinatal period and the antibiotic exposure later in life.

## Results

Fecal follow-up samples were received from 100 of the 150 study participants at the age of 12 months (Fig. 1a, b). Antimicrobial consumption data for the first 12 months were available from 99 infants (Fig. 1b).

### Perinatal antibiotics and the gut microbiota at the age of 1 year

**Fig. 2:**
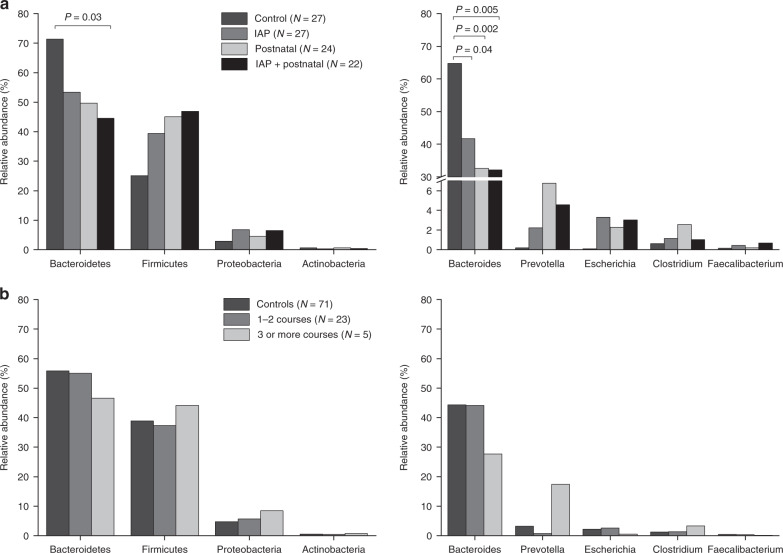
Relative abundances of the main bacterial phyla and genera of the gut microbiota at 1 year of age. The gut microbiota of the vaginally delivered term infants at 1 year, according to **a** perinatal antibiotic exposure and **b** antibiotic courses for common childhood infections. *P* values indicate post hoc comparisons (control vs. the various antibiotic groups).

**Fig. 3:**
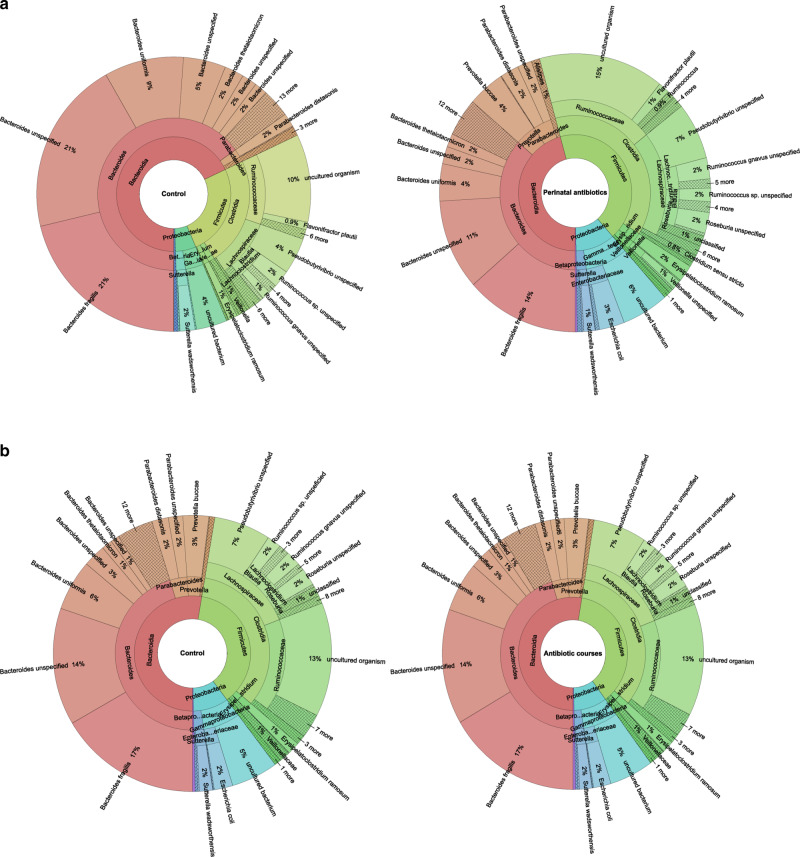
Krona charts showing the gut microbiota at one year of age. **a** After perinatal antibiotic exposure. **b** After courses of antibiotics. Summed results for the group in question and the control group.

Exposure to the perinatal antibiotics had an impact on the gut microbiota at the phylum level at 12 months of age (Table 2 and Figs. 2 and  3). The abundance of the Bacteroidetes phylum was highest in the control group (71%), followed by the IAP group (53%), the postnatal group (50%), and the IAP + postnatal group (45%) (Table 3 and Fig. 2a). The relative abundance of Bacteroidetes differed significantly between the control group and the perinatal antibiotics groups (71% vs. 49%), *P* < 0.001 (Table 2). When comparing all four groups with each other, the difference was statistically significant between the IAP + postnatal group and the control group (Table 3). The relative abundance of Firmicutes was highest in the IAP + postnatal group (47%), followed by the postnatal group (45%), the IAP group (39%), and the control group (25%) (Table 3 and Fig. 2a).

**Table 2:** Composition of the gut microbiome in vaginally delivered infants (*N* = 100) exposed to perinatal antibiotics and courses of antibiotics as compared with the control group after the first year of life.

	Control, *N* = 27, mean (SD)	Any perinatal antibiotics, *N* = 73, mean (SD)	95% CI of difference	*P* value^a^	No antibiotic courses, *N* = 71, mean (SD)	Antibiotic courses, *N* = 28, mean (SD)	95% CI^c^ of difference	*P* value^a^
Diversity^b^								
No. of OTUs	75 (21)	79 (22)	−14, 5.1	0.35	79 (22)	76 (22)	−6.4, 13	0.51
Shannon diversity index	3.0 (0.90)	3.1 (0.90)	−0.42, 0.39	0.95	3.0 (0.91)	3.1 (0.87)	−0.45, 0.34	0.78
Faith’s phylogenetic diversity index	7.5 (1.2)	8.1 (1.5)	−1.2, 0.03	0.06	7.9 (1.3)	8.0 (1.7)	−0.72, 0.55	0.80
Pielou’s evenness index	0.49 (0.13)	0.48 (0.12)	−0.05, 0.06	0.87	0.48 (0.13)	0.49 (0.12)	−0.07, 0.04	0.61
	Relative abundances (%)							
	Mean (SD)	Mean (SD)			Mean (SD)	Mean (SD)		
Phyla								
Bacteroidetes	71 (22)	49 (35)	10, 34	<0.001	56 (35)	53 (31)	−13, 17	0.76
Firmicutes	25 (22)	44 (32)	−30, −7.1	0.002	39 (32)	38 (30)	−13, 14	0.96
Proteobacteria	2.8 (4.6)	6.0 (12)	−6.5, 0.26	0.07	4.7 (12)	6.2 (9.3)	−6.4, 3.4	0.55
Actinobacteria	0.60 (1.8)	0.46 (0.77)	−0.38, 0.66	0.59	0.51 (1.3)	0.50 (0.73)	−0.50, 0.53	0.96
Tenericutes	0.06 (0.22)	0.004 (0.03)	−0.03, 0.14	0.21	0.02 (0.12)	0.03 (0.12)	−0.07, 0.04	0.56
Verrucomicrobia	0.06 (0.29)	0.52 (3.7)	−1.9, 0.95	0.52	0.05 (0.20)	1.3 (5.9)	−3.5, 1.0	0.27
Genera								
* Bacteroides*	65 (24)	36 (35)	16, 41	<0.001	44 (36)	41 (32)	−12, 19	0.69
* Clostridium*	0.63 (0.76)	1.6 (2.9)	−1.7, −0.23	0.01	1.2 (2.6)	1.7 (2.3)	−1.6, 0.65	0.41
* Faecalibacterium*	0.15 (0.25)	0.42 (0.80)	−0.48, −0.06	0.01	0.38 (0.77)	0.28 (0.52)	−0.22, 0.41	0.54
* Prevotella*	0.19 (0.62)	4.4 (15)	−7.8, −0.67	0.02	3.2 (12)	3.7 (16)	−6.4, 5.4	0.87
* Escherichia coli*	0.09 (0.14)	2.9 (10)	−5.2, −0.36	0.03	2.1 (10)	2.2 (6.2)	−4.1, 4.0	0.98
* Lactobacillus*	0.02 (0.04)	0.64 (4.6)	−2.4, 1.2	0.49	0.63 (4.7)	0.09 (0.26)	−1.2, 2.3	0.54
* Bifidobacterium*	0.47 (1.7)	0.26 (0.48)	−0.47, 0.90	0.53	0.34 (1.1)	0.27 (0.45)	−0.36, 0.50	0.75
* Blautia*	0.09 (0.25)	0.32 (0.98)	−0.47, 0.02	0.07	0.18 (0.62)	0.46 (1.3)	−0.79, 0.22	0.26
* Veillonella*	1.1 (3.4)	1.3 (3.9)	−1.9, 1.5	0.83	0.81 (2.0)	2.3 (6.3)	−4.0, 0.99	0.23

*CI* confidence interval.

^a^Independent samples *t* test was used to compare antibiotic groups with the control group.

^b^No. of OTUs and diversity indices could not be measured in one infant’s sample due to the small number of sequences.

**Table 3:** Composition of the gut microbiome in vaginally delivered infants (*N* = 100) exposed to various perinatal antibiotics as compared with the control group at 1 year of age.

	Control, *N* = 27, mean (SD)	IAP, *N* = 27, mean (SD)	Postnatal, *N* = 24, mean (SD)	IAP + postnatal, *N* = 22, mean (SD)	*P* value^a^ (ANOVA)	*P* value^b^ (post hoc)
Diversity^c^						
No. of OTUs	75 (21)	79 (26)	77 (18)	82 (20)	0.65	
Shannon diversity index	3.0 (0.90)	3.0 (0.93)	3.0 (0.87)	3.1 (0.92)	0.99	
Faith’s phylogenetic diversity index	7.5 (1.2)	8.0 (1.5)	8.1 (1.7)	8.1 (1.1)	0.32	
Pielou’s evenness index	0.49 (0.13)	0.48 (0.13)	0.48 (0.13)	0.49 (0.13)	0.99	
	Relative abundances (%)					
	Mean (SD)	Mean (SD)	Mean (SD)	Mean (SD)		
Phyla						
Bacteroidetes	71 (22)	53 (34)	50 (35)	45 (37)	0.007	0.03^d^
Firmicutes	25 (22)	39 (32)	45 (32)	47 (34)	0.02	
Proteobacteria	2.8 (4.6)	6.8 (13)	4.6 (8.8)	6.5 (15)	0.54	
Actinobacteria	0.60 (1.8)	0.35 (0.66)	0.61(0.97)	0.44 (0.67)	0.82	
Tenericutes	0.06 (0.22)	0.01 (0.05)	0.001 (0.003)	0.001 (0.002)	0.42	
Verrucomicrobia	0.06 (0.29)	0.07 (0.17)	0.07 (0.18)	1.6 (6.7)	0.78	
Genera						
* Bacteroides*	65 (24)	42 (36)	33 (33)	32 (37)	<0.001	0.04^e^ 0.002^f^ 0.005^g^
* Clostridium*	0.63 (0.76)	1.1 (1.6)	2.6 (4.3)	1.0 (1.6)	0.09	
* Faecalibacterium*	0.15 (0.25)	0.44 (0.63)	0.19 (0.27)	0.67 (1.2)	0.06	
* Prevotella*	0.19 (0.62)	2.2 (6.1)	6.8 (23)	4.6 (14)	0.09	
* Escherichia coli*	0.09 (0.14)	3.3 (11)	2.3 (6.0)	3.0 (14)	0.12	
* Lactobacillus*	0.02 (0.04)	1.5 (7.6)	0.06 (0.26)	0.18 (0.69)	0.41	
* Bifidobacterium*	0.47 (1.7)	0.19 (0.35)	0.31 (0.57)	0.27 (0.50)	0.77	
* Blautia*	0.09 (0.25)	0.04 (0.08)	0.41 (1.1)	0.55 (1.3)	0.10	
* Veillonella*	1.1 (3.4)	1.0 (2.9)	0.92 (2.3)	1.9 (6.0)	0.79	

*IAP* intrapartum antibiotic prophylaxis.

^a^Analysis of variance was used for comparisons between all groups.

^b^Post hoc test was used for comparisons between two groups. Statistically significant differences in the post hoc tests are reported in the table.

^c^No. of OTUs and diversity indices could not be measured in one infant’s sample due to the small number of sequences.

^d^Difference between the control group and the IAP + postnatal group.

^e^Difference between the control group and the IAP group.

^f^Difference between the control group and the postnatal group.

^g^Difference between the control group and the IAP + postnatal group.

*Bacteroides* was the most abundant genus in the samples; its relative abundance being highest in the control group, which differed significantly from the perinatal antibiotic groups (65% vs. 36%) (Table 2 and Fig. 2a). Comparing all four groups, statistically significant differences were seen between the control group (65%) and the IAP group (42%), between the control and the postnatal group (33%), and between the control and the IAP + postnatal group (32%) (Table 3 and Fig. 2a). The relative abundance of the genus *Clostridium* was lower in the control group than in the any perinatal antibiotic group, that is, IAP, postnatal, and IAP + postnatal groups combined (Table 2). Similarly, *Faecalibacterium* was less abundant in the control group than in the any perinatal antibiotic group (Table 2). The relative abundance of *Prevotella* was higher in the any perinatal antibiotic group than in the control group (Table 2). At the species level, the relative abundance of *Escherichia coli* was significantly lower in the control group than in the any perinatal antibiotic group (Table 2).

The number of OTUs did not differ between the control and perinatal groups (75 vs. 79), *P* = 0.35 (Table 2), and no differences in the microbial diversity were seen between these groups when measured in terms of Shannon diversity index or Pielou’s evenness index (Table 2). Likewise, Faith’s phylogenetic diversity index was 7.5 in the control group and 8.1 in the perinatal antibiotic group, but this difference was not statistically significant, *P* = 0.06 (Tables 2 and  4).

**Table 4:** Baseline characteristics of the participants (*N* = 100).

	Control, *N* = 27	Any perinatal antibiotic, *N* = 73
Birth weight (g), mean (SD)	3484 (465)	3602 (447)
Gestational age (day), mean (SD)	280 (8)	281 (8)
Birth mode, *N* (%)		
Vaginally delivered	27 (100)	73 (100)
Gender, *N* (%)		
Boy	14 (52)	37 (51)
Girl	13 (48)	36 (49)
Agpar, *N* (%)		
4–7	0 (0)	13 (18)
8–10	27 (100)	59 (81)
Exclusive breastfeeding at 6 months, *N* (%)^a^	19 (70)	48 (66)
Duration of the exclusive breastfeeding at 12 months (months), mean (SD)	5.5 (1.6)	5.1 (2.3)
Maternal smoking, *N* (%)	0 (0)	5 (7)
Paternal smoking, *N* (%)	3 (11)	17 (23)
*Lactobacilli* *reuteri* probiotic during perinatal period, *N* (%)	0 (0)	46 (63)
Probiotic use within 12 months, *N* (%)	16 (64)	43 (64)
Probiotic use (months), mean (SD)	7.1 (4.7)	8.2 (4.4)

^a^The missing data of the exclusive breastfeeding at 6 months has been counted as no exclusive breastfeeding.

### Courses of antibiotics and the gut microbiota at the age of 1 year

Courses of antibiotics were administered to infants in all the perinatal antibiotic groups (Fig. 1b). Altogether, 28 infants (28%) were exposed to a total of 44 such courses during the first 12 months (Fig. 1b and Table 1). β-Lactams (amoxicillin with or without clavulanic acid, cefuroxime, and cephalexin) were the most commonly used antibiotics comprising 35 (80%) of all the courses (Table 1). Macrolides (azithromycin and clindamycin) were used for five courses (11%) and sulfatrimethoprim for three of the courses (7%), with one antimicrobial course remaining unknown (Table 1). The median time between the last antibiotic course and the 12 months fecal sampling was 2 months in the control group (range 8, from 1 to 9) and the IAP group (range 4, from 1 to 5), and 3 months in the postnatal group (range 8, from 1 to 9) and the IAP + postnatal group (range 7, from 1 to 8) (Table 1).

Exposure to antibiotic courses during the first year of life was not associated with the infant’s gut microbiota at 1 year of age (Figs. 2b and 3 and Table 2). The relative abundance of Bacteroidetes was 47% in the group exposed to three or more courses of antibiotics, 55% in the group exposed to one or two courses, and 56% in the control group, the difference not being significant, *P* = 0.84 (Table 5, Fig. 2b). At the genus level, the relative abundance of *Bacteroides* was 28% in the group exposed to three or more antibiotic courses, 44% in the group exposed to one or two courses, and 44% in the control group, but again the difference was not statistically significant, *P* = 0.59 (Table 5 and Fig. 2b). Likewise, the relative abundance of *Clostridium* was 3.3% in the group exposed to three or more antibiotic courses, 1.3% in the group exposed to one to two antibiotics, and 1.2% in the control group, without the difference being statistically significant, *P* = 0.20 (Table 5 and Fig. 2b).

**Table 5:** Composition of the gut microbiome at 1 year of age in vaginally delivered infants (*N* = 99) according to their exposure to courses of antibiotics.

	No courses of antibiotics, *N* = 71, mean (SD)	1–2 courses, *N* = 23, mean (SD)	3 or more courses, *N* = 5, mean (SD)	*P* value^a^
Diversity^b^				
No. of OTUs	79 (22)	75 (20)	76 (30)	0.80
Shannon’s diversity index	3.0 (0.9)	3.1 (0.77)	2.9 (1.3)	0.85
Faith’s phylogenetic diversity index	7.9 (1.3)	8.0 (1.7)	8.0 (1.6)	0.97
Pielou’s evenness index	0.48 (0.13)	0.50 (0.11)	0.46 (0.19)	0.73
	Relative abundances (%)			
Phyla				
	Mean (SD)	Mean (SD)	Mean (SD)	
Bacteroidetes	56 (35)	55 (29)	47 (43)	0.84
Firmicutes	39 (32)	37 (28)	44 (39)	0.91
Proteobacteria	4.7 (12)	5.7 (9.7)	8.4 (7.9)	0.74
Actinobacteria	0.51 (1.3)	0.45 (0.57)	0.73 (1.3)	0.89
Tenericutes	0.02 (0.12)	0.03 (0.12)	0.06 (0.13)	0.74
Verrucomicrobia	0.05 (0.20)	1.6 (6.5)	0.03 (0.04)	0.43
Genera				
* Bacteroides*	44 (36)	44 (31)	28 (37)	0.59
* Clostridium*	1.2 (2.6)	1.3 (1.8)	3.3 (3.8)	0.20
* Faecalibacterium*	0.38 (0.77)	0.33 (0.56)	0.08 (0.07)	0.66
* Prevotella*	3.2 (12)	0.70 (2.3)	17 (39)	0.23
* Escherichia coli*	2.1 (10)	2.5 (6.8)	0.54 (0.90)	0.91
* Lactobacillus*	0.63 (4.7)	0.10 (0.29)	0.02 (0.04)	0.83
* Bifidobacterium*	0.34 (1.1)	0.24 (0.38)	0.39 (0.74)	0.91
* Blautia*	0.18 (0.62)	0.33 (0.95)	1.1 (2.3)	0.58
* Veillonella*	0.81 (2.0)	2.2 (6.7)	2.6 (4.9)	0.48

^a^One-way analysis of variance (ANOVA) was used for comparisons between all groups. No statistically significant differences were seen between the groups using the post hoc tests.

^b^No. of OTUs and diversity indices could not be measured in one infant’s sample due to the small number of sequences.

No differences were seen in the microbial diversity as measured by the Shannon index, Faith’s phylogenetic diversity index, or Pielou’s evenness index, nor did the number of OTUs differ between the antibiotic course groups (Table 5).

### Multivariate analysis of gut microbiome composition adjusted for different antibiotic exposures

Effects of the perinatal antibiotics and courses of antibiotics on the three main bacterial phyla, Bacteroidetes, Firmicutes, and Proteobacteria of the infant’s gut microbiota, were compared using the linear mixed-model analysis adjusted for different antibiotic exposure groups: IAP, postnatal, IAP + postnatal, and courses of oral antibiotics (any vs. none) (Table 6). Relative abundance of the Bacteroidetes was inversely associated with the exposure to IAP (*P* = 0.045), to postnatal antibiotics (*P* = 0.02), and to IAP + postnatal antibiotics (*P* = 0.005). Exposure to courses of oral antibiotics did not show a statistically significant effect (*P* = 0.68) (Table 6). Relative abundance of the Firmicutes was linearly associated with the postnatal group (*P* = 0.02) and the IAP + postnatal group (*P* = 0.01), but no associations were seen in the IAP group (*P* = 0.09) nor exposing to any courses of antibiotics (*P* = 0.99). No statistically significant associations were seen in the relative abundance of Proteobacteria and exposures to perinatal antibiotics or courses of antibiotics.

**Table 6:** Linear mixed-model analysis adjusted for four antibiotic exposure groups (IAP, postnatal IAP + postnatal, and oral antibiotic courses [any vs. none] after the perinatal period).

	Effect	*P* value	95% CI of difference
Bacteroidetes			
IAP	−0.18	0.045	−0.35, −0.004
Postnatal	−0.21	0.02	−0.39, −0.04
IAP + postnatal	−0.27	0.005	−0.45, −0.08
Antibiotic courses	−0.03	0.68	−0.17, 0.11
Firmicutes			
IAP	0.14	0.09	−0.02, 0.30
Postnatal	0.20	0.02	0.03, 0.36
IAP + postnatal	0.21	0.01	0.04, 0.39
Antibiotic courses	0.001	0.99	−0.13, 0.13
Proteobacteria			
IAP	0.04	0.17	−0.02, 0.10
Postnatal	0.02	0.55	−0.04, 0.08
IAP + postnatal	0.04	0.22	−0.02, 0.10
Antibiotic courses	0.02	0.50	−0.03, 0.06

The effects on the relative abundance of the three main bacterial phyla in gut microbiome composition at 1 year of age are shown. Effect indicates the linear effect estimate retrieved from the multivariate model.

*IAP* intrapartum antibiotic prophylaxis, *CI* confidence interval.

## Discussion

In this prospective, controlled cohort study among vaginally delivered infants, the impact of perinatal antibiotic exposure on the gut microbiota composition at the age of 1 year was greater than that of later courses of oral antibiotics. In the case of perinatal antibiotics, there appeared to be a dose-dependent impact, since the greatest difference at the phylum level was seen in infants exposed to both intrapartum antibiotics and intravenous postnatal antibiotics as compared with the control infants.

Several previous cross-sectional studies have reported an impact of IAP on the gut microbiota in vaginally delivered infants, with IAP being associated with a lower relative abundance of Bacteroidetes and higher relative abundance of Firmicutes at an age of 10 days^27^ and up to 3–6 months of age.^10,11^ At the genus level, IAP has been linked to lower relative abundance of *Bacteroides* and *Bifidobacterium*, and higher relative abundance of *Enterococcus* and *Clostridium*.^10,11,28,29^ Regarding the long-term impact of perinatal antibiotics, the evidence is more limited. One earlier study of 266 infants exposed to IAP showed that the relative abundance of *Bacteroides*, *Clostridium*, *Bifidobacterium*, and *Blautia* had decreased in the infants exposed to the most commonly used IAP, penicillin, by comparison with the non-exposed group at 1 year of age.^30^ In our previous study, at 6 months of age, perinatal antibiotic exposure was associated with a lower relative abundance of Bacteroidetes and *Bacteroides* as well.^11^ Thus, our present results at the age of 1 year are consistent with our present results at the age of 6 months. In the present study, we expand the earlier findings and show that perinatal antibiotic exposure appeared to have a long-term effect on the gut microbiota at the age of 12 months, which persisted even though infants had received courses of oral antibiotics for common childhood infections in particular between 6 and 12 months of age.

The previous evidence concerning oral antimicrobial consumption and the gut microbiota in children is limited, but in the case of adults, studies with 10–20 participants per group have reported alterations in the intestinal microbiota composition and decreased microbial diversity in groups exposed to vancomycin,^31^ clindamycin,^32^ and ciprofloxacin^32,33^ as compared with a control group. One large observational study among 200 daycare children showed that macrolide exposure was linked to an altered microbial composition, with a decrease in Actinobacteria and an increase in Bacteroidetes and Proteobacteria, but that the changes were less clear after the commonly used β-lactam antibiotics.^20^ In addition, in one earlier randomized, double-blinded and placebo-controlled study of 59 children, a 3-day oral exposure to azithromycin caused a reduced gut microbial diversity, richness, and relative abundance of Actinobacteria 14 days after the randomization, but these differences were no longer seen in the samples taken 13–39 months after the treatment.^34^ On the contrary, one recent study of 808 adolescents showed differences in the salivary microbiota according to lifetime antibiotic use,^35^ and in another observational study among 40 antibiotic-naive infants exposed to even a single amoxicillin course disrupted gut microbiota composition with a decreased relative abundance of *Bifidobacteria* persisting after 6 months.^36^

The majority of our infants were exposed to courses of β-lactam antibiotics and the fecal samples were taken on average 2 or 3 months after the exposure to antimicrobials. In our study, the impact of courses of antimicrobials was not as great as the perinatal antibiotics, and analogously to the two previous studies,^20,34^ a single course of an antimicrobial did not seem to have a clear long-term impact on the infant’s gut microbiota, but the number of children with three or more courses of antibiotics was too low to allow any conclusions to be made on the influence of repeated antibiotic exposure on the gut microbiota. However, the current research concerning the long-term impact of even a single course of oral antibiotics is contradictory and more research in this topic is highly needed.

The clinical significance of the gut microbiota changes described here is not yet known. Despite the considerable benefits of IAP in preventing GBS sepsis, some countries, including the United Kingdom, for example, have not adopted a routine screening for GBS in pregnant women due to concerns regarding overtreatment and a lack of knowledge of the long-term effects.^37^ Although no association between IAP and a higher early childhood BMI was found in one earlier study of 4825 mother/infant dyads,^38^ the observed gut microbiota changes warrant further epidemiological research into the long-term health effects of IAP and perinatal antibiotics, as has earlier been done in the case of the long-term outcome after Cesarean section (C-section).^39–43^ Interestingly, C-section has also been reported to result in decreased numbers of *Bacteroides* and higher numbers of *Clostridium* in the intestinal microbiota.^44–46^

Perinatal antibiotics were found here to be associated with a subsequent increase in *E. coli* in the gut microbiota at 1 year of age. Urinary tract infections (UTIs) are common infections in children and are most often caused by *E. coli*, which belongs to the phylum Proteobacteria.^47^ The relative abundance of *E. coli* in the gut microbiota has recently been shown to be associated with subsequent symptomatic *E. coli* UTI,^48,49^ and an increased relative abundance of Proteobacteria has been reported earlier to be more abundant in the gut microbiota in obese patients and those with Crohn’s disease.^50–52^

The strength of our study lies in the prospective cohort study design, which allowed comparisons to be made between the impacts of different exposures to perinatal antibiotics and courses of antimicrobials. Only vaginally delivered term infants were included in the present study, in order to reduce the confounding effects of prematurity and the mode of delivery. One possible confounding factor was that the infants exposed to postnatal antibiotics receiving *Lactobacilli reuteri* probiotic product during their stay in the hospital. However, in our earlier study, the early life difference in the relative abundance of *Lactobacilli* had vanished by the age of 6 months,^11^ and similarly, no such differences were seen in the present study. In addition, breastfeeding, maternal diet, solid foods, pets, and family size may modify or confound the observed effects of the antibiotics. The limitation of the present study was that we were not able to include them all in a multivariate model with the present sample size.

One limitation of the study is the small number of participants receiving three or more courses of antibiotics, which makes the statistical power of the findings insufficient for assessing the impact of repeated antibiotic courses. Also, since we did not have a fecal sample obtained shortly before and after the later courses of antibiotics, the short-term impact of these could not be studied. The methods used in the present study were limited to the relative gut microbiome composition created by the next-generation sequencing of bacterial 16S marker gene. In the future, functional metabolic profiling, proteomic analysis, metagenome sequencing for antimicrobial resistance genes, and characterization of the fungal microbiome, mycobiome, might add valuable insights to elucidate the antibiotic impact on the gut microbiome in children. Currently, there are limited data on the clinical correlates of the observed changes in the gut microbiome composition after perinatal antibiotic exposure.

In conclusion, this prospective cohort study shows that perinatal antibiotics, including IAP and postnatal intravenous antibiotics, have a marked, long-term impact on the composition of the infant’s gut microbiota at the age of 1 year. Since this impact did not vanish after the unexposed infants received courses of antibiotics for common childhood infections, the timing of the antibiotic exposure appears to be the critical factor for the changes observed in the gut microbiota.
